# Detection of BK virus in urine from renal transplant subjects by mass spectrometry

**DOI:** 10.1186/1559-0275-9-4

**Published:** 2012-04-26

**Authors:** Rebecca Konietzny, Roman Fischer, Nicola Ternette, Cynthia A Wright, Ben W Turney, Aron Chakera, David Hughes, Benedikt M Kessler, Chris W Pugh

**Affiliations:** 1 Centre for Cellular and Molecular Physiology, Henry Wellcome Building for Molecular Physiology, Nuffield Department of Medicine, University of Oxford, Roosevelt Drive, Oxford, OX3 7BN, UK; 2 Renal, Transplant and Urology Directorate, Churchill Hospital, Oxford, UK

**Keywords:** BK virus, Urine proteomics, Mass spectrometry, Renal transplant, Decoy cells

## Abstract

**Background:**

The diagnosis and management of BK virus (BKV) reactivation following renal transplantation continues to be a significant clinical problem. Following reactivation of latent virus, impaired cellular immunity enables sustained viral replication to occur in urothelial cells, which potentially leads to the development of BKV-associated nephropathy (BKVAN). Current guidelines recommend regular surveillance for BKV reactivation through the detection of infected urothelial cells in urine (decoy cells) or viral nucleic acid in urine or blood. However, these methods have variable sensitivity and cannot routinely distinguish between different viral subtypes. We therefore asked whether mass spectrometry might be able to overcome these limitations and provide an additional non-invasive technique for the surveillance of BKV and identification of recipients at increased risk of BKVAN.

**Results:**

Here we describe a mass spectrometry (MS)-based method for the detection of BKV derived proteins directly isolated from clinical urine samples. Peptides detected by MS derived from Viral Protein 1 (VP1) allowed differentiation between subtypes I and IV. Using this approach, we observed an association between higher decoy cell numbers and the presence of the VP1 subtype Ib-2 in urine samples derived from a cohort of 20 renal transplant recipients, consistent with the hypothesis that certain viral subtypes may be associated with more severe BKVAN.

**Conclusions:**

This is the first study to identify BK virus proteins in clinical samples by MS and that this approach makes it possible to distinguish between different viral subtypes. Further studies are required to establish whether this information could lead to stratification of patients at risk of BKVAN, facilitate distinction between BKVAN and acute rejection (AR), and ultimately improve patient treatment and outcomes.

## Background

BK virus, a member of the polyomavirus family, infects the majority of the population during childhood [1]. In most cases infection is asymptomatic; however, BKV persists in the urothelial tract with intermittent reactivation occurring throughout life [2,3]. In the presence of immunosuppression sustained viral replication may occur due to escape of the endogenous virus from immune control or in renal transplant recipients through co-infection with virus of donor origin [4,5]. If viral replication remains uncontrolled, lytic destruction of infected cells occurs, eventually disturbing kidney function, and resulting in the characteristic biopsy appearances of BK-virus associated nephropathy (BKVAN) [6]. Distinguishing between acute rejection and BKVAN is important, because although the histological appearances may be similar, graft rejection necessitates increased immunosuppression, whereas control of BK viral replication requires immunosuppression reduction. Overall, improving the subject’s clinical status requires care in achieving a balanced immunosuppressive regimen, particularly as there is an inevitable lag between changes in drug therapy and clinical response.

Screening for BK virus in kidney transplant recipients is usually carried out by the detection of virally infected cells in urine or viral nucleic acid in urine or blood [7]. Urine cytology is often used as a screening test for active viral infection by looking for decoy cells; urothelial cells with an enlarged nucleus containing a single large basophilic intra-nuclear inclusion [8]. However, the sensitivity and specificity of decoy cell measurement is debated [9-13]. BK viral DNA in urine or plasma samples can be measured to determine viral load, but detection depends on the primer used and does not usually distinguish the subtypes [12]. However, despite monitoring the transplant recipient’s kidney function, decoy cell counts, and viral load in plasma / urine samples, characterizing the degree of BKVAN remains a challenge. To overcome this, a number of other techniques are under evaluation. Serological testing is problematic due to the high background level of sero-positivity, which is in itself insufficient to prevent disease. Assays for the assessment of BKV-specific T-cell responses remain experimental [14]. Urine electron microscopic detection of viral aggregation has been reported to be highly sensitive and specific but is complex to perform and not used routinely [15]. Most recently BK viral genotyping by high-resolution melting analysis has been described [16]. Despite all these options repeated renal biopsies are sometimes required. However, even with this invasive approach evaluation remains difficult due to patchy infection and the overlap in histological appearances between BKVAN and acute rejection.

BK viruses have evolved into four serologically distinct subtypes (I–IV). Furthermore, subtype I can be divided into Ia, Ib-1, Ib-2 and Ic. The genome sequence of Ia (Dunlop strain) was first described by Seif et al., 1979 [17]. DNA isolated from urine of BKVAN subjects has shown mutations encoding amino acid substitutions throughout the highly variable major capsid viral protein VP1; the protein responsible for attachment to and subsequent infection of the host cell via an α-(2,3)-linked sialic acid on N-linked glycoproteins [18-20]. Longitudinal analyses of kidney biopsies have also shown changes in the VP1 sequence within individual subjects [21]. Although correlations between BKV subtypes and clinical outcome remain controversial [22], some previous studies indicate that certain subtypes may cause more complications than others [23].

Non-invasive routes to analyze the presence of different BK viral subtypes may enhance our understanding of the general pathology of BK viruses and provide new entry points to address the problem of BKVAN.

In the present pilot study, we developed a mass spectrometry-based method to identify and characterize BKV proteins in urine samples from renal transplant subjects. Our results demonstrated the identification of BK virus subtypes and provided evidence of co-infection in several patients.

## Results and discussion

In this pilot clinical study urine samples from 20 renal transplantation recipients (Table 1) were analyzed for BK viral protein content via mass spectrometry. In our unit decoy cell assessment is routinely and cost effectively used to detect BK viral infection [24]. Fourteen of these patients were selected because of current or recent decoy cell positivity, whereas the other six patients were selected as negative controls on the basis that they had never had decoy cells detected in their urine. Serum creatinine levels were assayed to assess kidney function at the time of collection and averaged 143.5 μmol / L with a range of 73–364 μmol / L. The normal range for creatinine levels observed in people with healthy kidney function in our hospital is between 54–145 μmol /L.

**Table 1:** Clinical Data of study subjects

**Subject**	**Age**	**Gender**	**Serum Creatinine Level (μmol/L)**	**Days post transplant**	**Vasudev Index**[25]
1	60	M	112	32	5.25
2	43	F	113	433	7.5
3	53	F	95	382	6.5
4	52	M	163	209	2.5
5	41	F	364	1597	7
6	29	M	108	191	3
7	62	M	230	63	6.5
8	48	M	118	119	2.5
9	72	M	200	246	2.5
10	47	M	112	238	4
11	32	F	113	459	2
12	47	F	163	224	10
13	72	M	139	63	4
14	58	F	73	340	3
15	42	M	164	235	7.8
16	49	M	122	333	4.5
17	54	M	129	994	2.5
18	66	M	135	344	5
19	49	F	99	21	6
20	39	M	118	142	3.5

This study was based on 20 subjects who had received renal transplants for end-stage renal failure. They had an average age of 50.75 and a range of 29-72 years (Table 1); 65% were male. The average serum creatinine level was 143.5 µmol / L and the range was 73-364 µmol / L at the time point of collection. The average collection day post-transplant was 333.25 days and the range was 21-1597 days. Immunosuppressant therapies were defined using the Vasudev Index [29].

**Figure 1:**
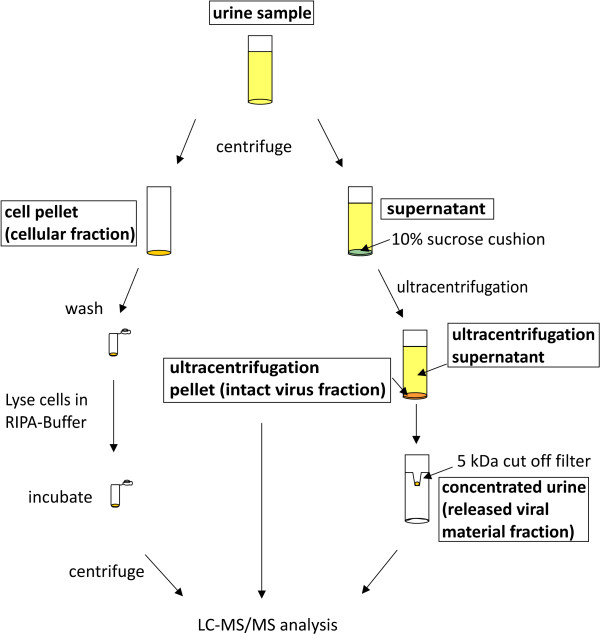
**Centrifugation-based separation and enrichment workflow of urine samples.** Urine samples were separated via centrifugation into three fractions: a cellular fraction containing epithelial cells pelleted by centrifugation, an intact virus fraction generated via ultracentrifugation through a 10% sucrose cushion and the ultracentrifugation supernatant was concentrated by a 5 kDa cut off filter to produce a fraction containing released viral material. Proteins from all fractions were methanol / chloroform precipitated and in-solution digestion was carried out followed by LC-MS/MS analysis.

Initial studies on unfractionated urine did not lead to the identification of BK viral peptides by mass spectrometry (results not shown). A differential centrifugation-based separation and enrichment protocol was therefore developed (see Figure 1) which did allow identification of a variety of viral peptides in the different fractions. Although slightly complex, this workflow allowed distinction between viral material in urinary cells (cellular fraction) and within the supernatant between intact virus (intact virus fraction) and viral material released following cell lysis (released viral material fraction). The approach described may allow distinction between the phases of infection in which epithelial shedding is prominent, effective on-going viral replication is occurring and the destructive aspects of cellular infection remain unchecked respectively.

Fractionated urine samples were subjected to analysis by LC-MS/MS tandem mass spectrometry and the observed peptides were searched against a customized database where BK viral protein sequences from different subtypes were combined with a human database. One limitation of mass spectrometry is a bias towards the detection and identification of the most abundant analyte in a given sample. It is therefore not surprising that the highest viral protein coverage in samples assessed to be BKV positive by decoy cell count was of the BKV protein VP1, a high copy number constituent of viral particles. This viral protein was mainly found in the released viral material fraction, perhaps reflecting the virus’ lytic properties, but it was also identified in the cellular fraction of some of the BKV positive samples, despite the more complex protein content of this fraction. In contrast, the Large T protein was only detectable in some of the released viral material fractions (unpublished data).

**Figure 2:**
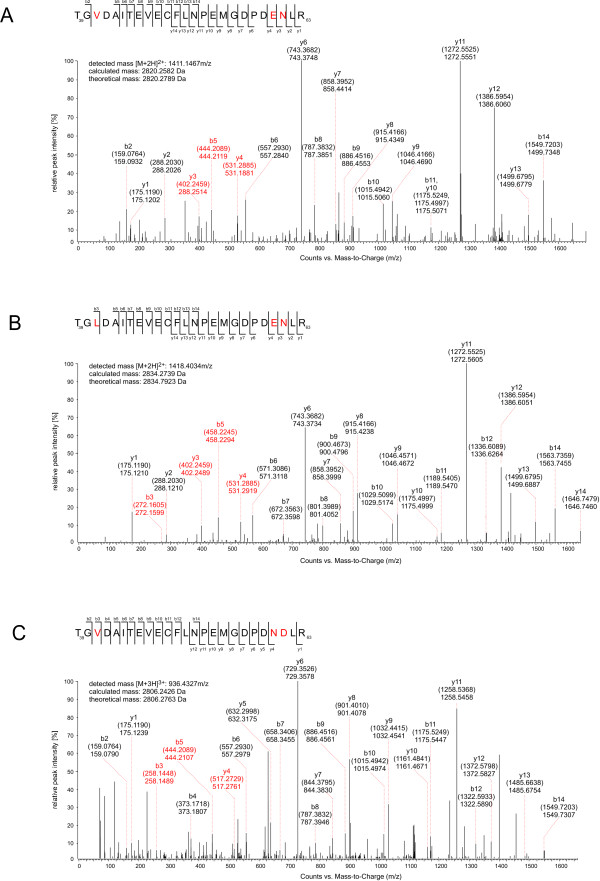
**Peptide analysis by LC-MS/MS allowing differentiation between BKV-VP1 subtypes.** MS/MS analysis of a tryptic peptide corresponding to aa 39-63 of VP1 distinguished between subtypes (Figure 2A-C). Each panel shows the amino acid sequence for the relevant viral subtype detected in a clinical isolate. Measured and predicted masses (in brackets) of the b and y fragment ion series are shown within each spectrum. Amino acids that differ between the subtypes and the corresponding b and y fragment ions are indicated in red. **(A)** The first peptide (TGVDAITEVECFLNPEMGDPDENLR) corresponds to the subtypes Ia, Ib-1 and Ic. **(B)** The second peptide (TGLDAITEVECFLNPEMGDPDENLR) is specific for subtype Ib-2, whereas **(C)** the last peptide (TGVDAITEVECFLNPEMGDPDNDLR) is observed in subtype IV only. Subtype Ib-2 (panel B) is distinguishable from the other subtypes by the presence of a leucine residue instead of a valine residue at position 41, resulting in a corresponding mass difference of +14 Da in the b ion series from b3 onwards. Subtype IV (panel C) differs from all subtype I variants (panels A and B) by the presence of asparagine and aspartate instead of glutamate and asparagine at positions 60 and 61 resulting in a corresponding mass difference of +14 Da in the y ion series from y4 onwards.

The BK viral VP1 protein sequence is known to be highly variable between viral subtypes. To detect the different BK virus subtypes in the renal transplant recipients the BKV positive samples were further analyzed with respect to VP1 sequence coverage, the number of peptides found derived from this protein and links between the observed peptides and the known viral subtypes. Although the BKV VP1 protein (Swiss Prot Acc No: P03088) shares similarities between JC (P03089), SV40 (P03087), Merkel cell polyomavirus (B6DVZ3), WU polyomavirus (A5HBD5) and KI polyomavirus of 78.2%, 81.5%, 43.9%, 28.8% and 28.1%, respectively the peptide fragment presented in Figure 2 is unique to BKV VP1. In this peptide (aa 39–63) amino acid substitutions at 41, 60 and 61 distinguish between groups containing subtypes Ia, Ib-1 and Ic, subtype Ib-2 or subtype IV (Figure 2). Of the thirteen BKV positive samples, analysis of the most abundant peptides detected showed four of these individuals were infected with the Ib-2 strain, two individuals were infected with strain IV, one subject was Ib-1 positive and one subject was infected with the Ic subtype. Peptides from subtype I were detected in the remaining five infected individuals, but it was not possible to classify their infections into sub-group strains (e.g. Ia, Ib-1, Ic) due to lack of sequence coverage (Table 2).

**Table 2:** BKV VP1 subtypes in renal patients detected by MS

**Patient**	**decoy cells**	**qPCR titer**	**dominant subtype**	**Sequence coverage VP1**	**aa**	**unique Peptides detected from dominant infection**	**co-infection subtype**	**Sequence coverage VP1**	**aa**	**unique Peptides detected from co-infection**
3	> 10	negative	Ic	21%	40–64	K.TGVDAITEVECFLNPEMGDPDENLR.G				
					173–195	K.YPEGTITPKNPTAQSQVMNTDHK.A				
5	>10	3.1×10^5^	Ib-1	74%	171–181	R.TKYPDGTITPK.N	IV	40%	94–135	R.IPLPNLNEDLTCGNLLMWEAVTVKTEVIGITSMLNLHAGSQK.V
7	>100	4.1×10^3^	Ib-2	60%	40–64	K.TGLDAITEVECFLNPEMGDPDENLR.G	Ib-1	51%	171–181	R.TKYPDGTITPK.N
8	>10	2.0×10^4^	IV	55%	40–64	K.TGVDAITEVECFLNPEMGDPDNDLR.G	Ic	37%	334–349	R.VFDGTEKLPGDPDMIR.Y
9	>100	1.4×10^3^	Ib-2	22%	40–69	K.TGLDAITEVECFLNPEMGDPDENLRGFSLK.L				
10	<5–10	not done	Ib-2,Ic	20%	70–84	K.LSAENDFSSDSPERK.M				
11	>10	negative	Ia,Ib-1	46%	40–64	K.TGVDAITEVECFLNPEMGDPDENLR.G	IV	29%	94–135	R.IPLPNLNEDLTCGNLLMWEAVTVKTEVIGITSMLNLHAGSQK.V
					221–256	R.YFGTFTGGENVPPVLHVTNTATTVLLDEQGVGPLCK.A				
13	>100	2.2×10^4^	Ib-2	72%	40–64	K.TGLDAITEVECFLNPEMGDPDENLR.G	IV	53%	350–360	R.YIDRQGQLQTK.M
14	negative	negative	Ib-2	35%	40–64	K.TGLDAITEVECFLNPEMGDPDENLR.G				
15	>10	2.4×10^4^	IV	64%	40–64	K.TGVDAITEVECFLNPEMGDPDNDLR.G	Ic	42%	334–349	R.VFDGTEKLPGDPDMIR.Y
16	>10	not done	Ia, Ib-1	56%	201–215	K.NNAYPVECWVPDPSR.N	IV	23%	94–135	R.IPLPNLNEDLTCGNLLMWEAVTVKTEVIGITSMLNLHAGSQK.V
17	<5	5×10^1^		not detected		not detected				
18	>10	1.9×10^4^	Ia, Ib-1,Ic	19%	40–69	K.TGVDAITEVECFLNPEMGDPDENLRGFSLK.L				
19	>10	negative	Ia, Ib-1	64%	40–64	K.TGVDAITEVECFLNPEMGDPDENLR.G	IV	44%	40–64	K.TGVDAITEVECFLNPEMGDPDNDLR.G
					221–256	R.YFGTFTGGENVPPVLHVTNTATTVLLDEQGVGPLCK.A				

For those subjects in whom peptides from VP1 were detected the number of decoy cells, the viral load in serum samples, the dominant viral subtype identified, and percent sequence coverage of VP1 protein are listed. Serum samples were collected within 2 weeks of the urine sample (except for patient 9 where the interval was 4 months). The peptides identified that defined the dominant viral subtype (based on greater BKV VP1 protein sequence coverage) are shown. In some cases the presence of two peptides gave evidence for a precise subtype specification. Further, peptides demonstrating the presence of co-infection with a different viral subtype are also shown where co-infection was observed. Residues highlighted in bold differ between the subtype groups Ia,Ib-1, Ic or Ib-2 or IV.

In eight of the thirteen BK positive individuals (Table 2), additional peptides were found that had alternative amino acid substitutions in the sequences that defined the identified subtype. This data clearly indicated the presence of an additional strain in these subjects, i.e. evidence of co-infection. While the majority of primary infections were due to subgroups of the subtype I strain (11/13), co-infections tended to be with subtype IV (5/8) rather than a different subtype I subgroup (1/8). In the two patients with primary subtype IV infection, co-infection was with subtype 1 strains.

To determine whether a correlation existed between dominant subtypes and severity of infection, cytological analysis of decoy cells was compared to the data obtained by mass spectrometry. VP1 was detected in all samples with more than ten decoy cells (n = 11) (Table 2), one sample with a decoy count of 5–10 and one sample where the decoy count was negative at the time of mass spectrometry analysis but had been positive for decoy cells on previous visits (subject 14). No virus material was detected by MS in subject 17, who had a decoy cell count under five. This may also have been due to the fact that a smaller volume of sample (50%) was available from this subject for the mass spectrometry analysis. No viral protein was detected by mass spectrometry in any of the six urine samples from patients who had been negative for decoy cells throughout their transplant course. In subject 14 VP1 was detected in the released viral material fraction, suggesting that viral material was still being produced although not leading to effective production of intact virus or the shedding of infected epithelial cells. This observation suggests that MS may be a more sensitive measurement of ongoing viral replication than decoy cell assessment and raises the interesting possibility that not only the presence of viral peptides, but the fraction(s) in which they are found, may have a bearing on the patient’s response to changes in immunosuppression and ultimate clinical outcome.

Interestingly, the three individuals that were Ib-2 positive demonstrated the highest number of decoy cells (>100), indicating that renal pathology may differ between different viral subtypes. It would be interesting to investigate this observation further in a larger cohort. There was no obvious correlation between co-infection with multiple strains and decoy cell number. We also investigated the question of the correlations between viral load within the serum sample, detected decoy cells in positive urine samples and the mass spectrometric data (Table 2). The patient with the lowest detected decoy cell load (<5) also had the lowest viral load within serum (5×10^1^) and no detectable VP1 peptide by mass spectrometry. However, overall the viral loads observed in positive serum samples were from 1.4×10^3^–3.1×10^5^ with no clear correlation to the decoy cell status (>10 or >100) of the patient or the BKV VP1 protein coverage identified by mass spectrometry of urine samples.

A limitation of this study relates to the use of databases which are comprised of known viral protein sequences. Peptides produced from novel mutant viral proteins will not match sequences present in the database and will be ignored. PCR based techniques face similar problems since the choice of primers based on known viral sequences may fail to distinguish between variants or limit detection of unknown variants. These factors may lead to an underestimation of the extent to which clinical outcome of patients varies because of differences in the virus subtype(s) present.

A further level of heterogeneity arises from post-translational modification of viral proteins, which cannot be detected at all by nucleic acid based approaches. Such modifications of BKV structural proteins have been identified by mass spectrometry in a BKV cell culture model [26]. In our clinical isolates we also detected modifications consistent with previous observations, as well as other yet unreported ones (unpublished data). However, the significance of these modifications in the context of the pathology of BKV and BKVAN remains to be determined. Simple analysis of our data indicates that in comparison with detection of any decoy cells in the same urine sample our mass spectrometric approach has a sensitivity of 93%, a specificity of 87.5%, a positive predictive value of 93% and a negative predictive value of 87.5%. However, it is arguable that these figures underestimate the utility of this assay since the results from subject 17 may have arisen for technical reasons and the results from subject 14 are more likely to represent a false negative decoy cell result than a false positive mass spectrometry result. Our approach yields information about the viral subtypes present, co-infection and the fractions in which viral peptides were detected and thus may provide clinically useful information that goes well beyond a simple diagnosis of BK viral infection.

## Conclusions

BKV infection following renal transplantation remains a major cause of graft loss and an important clinical problem [27]. As there is no antiviral drug available, the cornerstone of management is early detection of infection and cautious adjustment of immunosuppressive treatment such that the immune system is able to combat the disease without provoking kidney graft rejection [28]. Although decoy cell detection provides some estimate of viral load and activity, it neither identifies the presence of different viral subtypes which may vary in their pathogenicity [29] nor provides any direct estimate of production of intact virus or virus induced cell lysis. In contrast, in this pilot study, we have developed a mass spectrometry approach that can detect viral peptides derived from lysed cells, intact virus, shed epithelial cells and identify the presence of different subtypes providing novel leads into disease outcome.

A variety of factors (viral and non-viral) most certainly contribute to disease severity. However, we provide preliminary evidence that suggests that the subtype Ib-2 infection appears to be related to an elevated number of decoy cells present in the urine and thus clinical phenotype.

Overall, we believe these results should stimulate a longitudinal prospective study on a larger cohort to assess correlations between the presence of different viral subtypes, the fractions in which viral peptides are present and the severity and course of clinical infection as measured by effects on graft function.

## Methods

### Urine sample collection and preparation

20 urine samples were collected from recipients of kidney transplant (Renal, Transplant and Urology Directorate, Churchill Hospital, Oxford, U.K.). The average age of the subjects was 50.75 years and the range was 29–72 (Table 1). 35% of patients were female. The average collection day post-transplant was 333.25 days with a range of 21–1597 days. Individual immunosuppressive therapies at the time of urine sampling are listed using the immunosuppressive indices defined by Vasudev et al (Table 1) [25]. The samples were kept frozen at −20 degrees until analysis.

A differential centrifugation and filtration protocol was developed to enrich for viral material. Briefly, 15–35 ml of urine was centrifuged at ~ 230 g for 10 min at 4°C in a Beckman centrifuge (CS-6R) to pellet cells. After centrifugation, the cell pellet (cellular fraction) was washed twice with 1 ml of PBS and resuspended in 100 μl of RIPA-Buffer (50 mM Tris, 150 mM NaCl, 1 mM EDTA, 1% NP-40, 0.5% DOC, 0.1% SDS, containing protease inhibitor cocktail (Roche)). After incubation on ice for 1 hour cells were centrifuged at 16,200 g for 10 min at 4°C and the supernatant was subjected to methanol / chloroform extraction [30] and resuspended in 100 μl 6 M Urea in 100 mM Tris Buffer, pH 7.4. Samples were than digested in solution as described below. The cell-free viral material was concentrated from the supernatant using a 10% sucrose cushion in PBS by ultracentrifugation at ~ 43,000 g for 3 h at 4°C using a SW 28 rotor (Beckmann Coulter) in a Beckmann Optima XL-90. The ultracentrifugation pellet (intact virus fraction) was resuspended in 100 μl 6 M Urea in 100 mM Tris Buffer and subjected to in-solution digestion. Finally, the supernatant was concentrated using a 5 kDa cut off filter (Vivaspin, Sartorius), and this concentrated material (released viral material fraction) was subjected to methanol / chloroform extraction [30], resuspended in 100 μl 6 M Urea in 100 mM Tris Buffer and subjected to in-solution digestion. In-solution digestion of the prepared fractions was carried out as follows: The protein mixture was reduced by the addition of 1 μmol of dithiothreitol (DDT) for 60 min at room temperature and then alkylated by the addition of iodoacetamide (IAM) (4 μmol) for 60 min at room temperature. To consume any unreacted iodoacetamide, 4 μmol of dithiothreitol was added to the protein mixture and incubated for another 60 min at room temperature. The reaction mixture was diluted with 775 μl of MilliQ water and digestion with 20 ng Trypsin (Promega) was carried out overnight at 37°C. A Sep-Pak®Plus C18 column (Waters) purification was carried out to desalt and concentrate peptides following the manufacturer’s instructions, followed by solvent evaporation to dryness. Samples were resuspended in 20 μl 2% acetonitrile, 0.1% formic acid and kept at −20°C until mass spectrometry analysis.

### Analysis by tandem mass spectrometry

Peptides were analysed by nano-LC-MS/MS using a large capacity Chip (II), 150 mm 300 Å C18 analytical column with a 160 nL trap column (Agilent), coupled to an Agilent 6520 quadrupole time-of-flight (Q-Tof) tandem mass spectrometer. Chromatographic separation of small peptides was performed using a 76 min gradient from 2% acetonitrile, 0.1% formic acid to 98% acetonitrile, 0.1% formic acid at a flow rate of 600 nl / min. Data were acquired in MS and MS/MS mode and an inclusion list was generated to look for specific viral proteins ( Additional file 1: Table S1). Raw data was converted into the Mascot generic file format (mgf) with Masshunter software (Agilent, version B.03.01) and searched against a customized database where 84 BK viral protein sequences retrieved from trEMBL/SwissProt were combined with the UniProtKB / Swiss-Prot (20,287 human sequences) using an in-house Mascot server (Matrix Science, Version 2.3). The accession numbers of the BKV VP1 unique sequences are: spP03088 (subtype Ia), trQ65613 (subtype Ib-1), trQ0PCN6 (subtype Ib-2, E82D, V362L), trA8QZN3 (subtype Ib-2, E82D, V362L, R340L), trQ65620 (subtype Ic), trQ0PCM5 (subtype IVa, E77D) and trB6VQF5 (subtype IVc) using subtype classification based on reference [17].

### Decoy cell determination

15 ml of urine was centrifuged at 2000 rpm for 5 min. The pellet was resuspended in 1 ml of diluted Sedfix (10% Sedfix (Surgipath Europe Ltd, Cambridgeshire) in 45% industrial methylated spirits (IMS) (Surgipath Europe Ltd, Cambridgeshire) and deionised water). Slides of fixed cells were made by centrifuging ten drops of the cell suspension for 9 min at 850 rpm in a cytospin 3 apparatus (Shandon Inc., Pittsburgh, Pa). The air-dried cytospin slides underwent the following staining procedure: slides were immersed five times in 100% industrial methylated spirit (IMS) (Surgipath Europe Ltd, Cambridgeshire), and then immersed in 70% IMS and washed for 30 sec in water. Slides were incubated for 3 min in Gill’s haematoxylin I (Surgipath Europe Ltd, Cambridgeshire) and were then washed in water for 30 sec. Slides were then dipped twice in 1% hydrochloric acid (in IMS) and washed for 2 min in water followed by five washes in 70% and 100% IMS, respectively. After an incubation in OG-6 (Orange G, Surgipath Europe Ltd, Cambridgeshire) for 2 minutes, the slides were washed ten times in 100% IMS. The slides were incubated in EA-50 for 3 minutes and then washed again in 100% IMS. Clearing was done in two changes of Clearene (Surgipath Europe Ltd, Cambridgeshire) and slides were mounted in DPX (Surgipath Europe Ltd, Cambridgeshire). A light microscope was used to scan each slide methodically for BKV positive cells (decoy cells). The number of decoy cells identified was recorded as 0, <5, 5–10,> 10 or > 100.

### Quantitative real time PCR

The assessment of BK viraemia by quantitative real time PCR was performed at the Health Protection Agency’s South West Regional Laboratory in Bristol, UK as part of the routine clinical care of these patients.

## Abbreviations

BKV, BK virus; BKVAN, BK virus associated nephropathy; LC-MS/MS, Liquid chromatography tandem mass spectrometry; VP1, Major capsid protein VP1; MS, Mass spectrometry.

## Competing interests

The authors declare that they have no competing interests.

## Authors’ contribution

RK carried out the experimental procedures, analyzed the MS data and drafted the manuscript. RF operated the MS instrument and participated in the analysis of the MS data. NT participated in the design of the fractionation protocol. AC and DH provided clinical data. DH collected clinical samples and analyzed the samples for the presence of decoy cells. CW, BT participated in the design of the study and discussed experimental procedures. BK, CW participated in the analysis of the MS data and assisted in drafting the manuscript. CP conceived of the study, coordinated and helped to finalize the manuscript. All authors read and approved the final manuscript.

## Supplementary Material

Additional file 1 **Table S1.**VP1 derived tryptic peptides for detection by tandem mass spectrometry. Click here for file
